# Energy shifts induce membrane sequestration of DraG in *Rhodospirillum rubrum* independent of the ammonium transporters and diazotrophic conditions

**DOI:** 10.1093/femsle/fny176

**Published:** 2018-07-13

**Authors:** Helen Wang, Dominik Waluk, Ray Dixon, Stefan Nordlund, Agneta Norén

**Affiliations:** 1 Department of Medical Biochemistry and Microbiology, Uppsala Biomedicinska Centrum, Husarg.3, S-75237 Uppsala, Sweden; 2 Department of Biochemistry and Biophysics, Arrhenius Laboratories, Stockholm University, Svante Arrhenius v. 16C, Stockholm S-10691, Sweden; 3 Department of Molecular Microbiology, John Innes Centre, Norwich NR47 UH, UK

**Keywords:** metabolic regulation, nitrogenase, DraG, energy switch-off

## Abstract

Metabolic regulation of *Rhodospirillum rubrum* nitrogenase is mediated at the post-translational level by the enzymes DraT and DraG when subjected to changes in nitrogen or energy status. DraT is activated during switch-off, while DraG is inactivated by reversible membrane association. We confirm here that the ammonium transporter, AmtB1, rather than its paralog AmtB2, is required for ammonium induced switch-off. Amongst several substitutions at the N100 position in DraG, only N100K failed to locate to the membrane following ammonium shock, suggesting loss of interaction through charge repulsion. When switch-off was induced by lowering energy levels, either by darkness during photosynthetic growth or oxygen depletion under respiratory conditions, reversible membrane sequestration of DraG was independent of AmtB proteins and occurred even under non-diazotrophic conditions. We propose that under these conditions, changes in redox status or possibly membrane potential induce interactions between DraG and another membrane protein in response to the energy status.

## Teaser

DraG, an enzyme required for metabolic regulation of nitrogenase in *Rhodospirillum rubrum*, is regulated by reversible membrane sequestration during energy depletion, independent of diazotrophic conditions and presence of ammonium transporters.

## INTRODUCTION

*Rhodospirillum rubrum* is a diazotrophic, photosynthetic α-proteobacterium that forms a highly invaginated membrane structure induced by anaerobic growth in the light or by microaerobic growth under reducing conditions in the dark (Grammel and Ghosh 2008; Carius, Henkel and Grammel 2011; Carius, Hadicke and Grammel 2013; Grammel, Gilles and Ghosh 2003). Under all diazotrophic conditions, nitrogenase, which catalyses the reduction of nitrogen, has to be protected against high oxygen concentrations and supplied with reductant for the conversion of dinitrogen to ammonia.

Biological nitrogen fixation is a tightly regulated process in diazotrophic bacteria, regulated at a transcriptional and in some free-living diazotrophs at the post-translational level. The latter involves the action of two enzymes, DraG (dinitrogenase reductase activating glycohydrolase) and DraT (dinitrogenase reductase ADP-ribose transferase) that act in a reciprocal manner (Liang *et al.*^1991^; Lowery, Lehman and Ludden 1986; Masepohl, Krey and Klipp 1993). When subjected to an external ‘switch-off’ signal, active DraT catalyses ADP-ribosylation of dinitrogenase reductase (encoded by *nifH*), thereby interfering with electron transfer to dinitrogenase by preventing the interaction with dinitrogenase reductase. When the signal is removed, active DraG hydrolyses the N-glycosidic bond to the ADP ribosyl moiety and thereby restores nitrogenase activity (Ludden 1994). Addition of ammonia or decreasing the energy available to a nitrogen-fixing culture results in a fast response where DraG is inactivated. The intracellular signals leading to this regulation are not completely understood, particularly in the case of energy switch-off. However, our understanding of the ammonia-induced regulation and the players/proteins involved in this regulatory system has increased considerably in recent years. In both *R. rubrum* and *Azospirillum brasilense*, DraG regulation has been shown to be mediated by a reversible membrane association, inactivating DraG (Huergo *et al.*2006b; Wang *et al.*^2005^). When the ammonia has been metabolised DraG is released into the cytosol, regaining its activity.

In *R. rubrum,* ammonia-induced DraG association to the membrane is lost in an *amtB1* mutant resulting in a constitutively active DraG and loss of dinitrogenase reductase (NifH) modification (Wang *et al.*^2005^). In many organisms, PII signal transduction proteins control the activity of the ammonium transporter AmtB in response to nitrogen availability (Huergo *et al.*^2012^). Under nitrogen excess, the non-modified PII protein forms a stoichiometric complex with AmtB at the membrane, thus blocking ammonium transport. In proteobacteria, the PII-AmtB interaction is responsive to post-translational uridylylation by the GlnD uridylyl transferase/uridylyl removing enzyme, which is regulated by the intracellular concentration of glutamine. Under nitrogen-deficient conditions, the PII proteins are uridylylated by GlnD, resulting in the dissociation of PII from AmtB and a release into the cytosol. This regulated interaction between PII and AmtB in response to nitrogen availability also controls the association of DraG with the membrane as shown in *A. brasilense,* where the PII protein, GlnZ is required for DraG membrane sequestration upon sudden increases in ammonia concentration*,* in addition to the requirement for AmtB (Huergo *et al.*2006b). This suggests formation of an AmtB–GlnZ–DraG ternary complex at the membrane in response to ammonium shock. This model is supported by the observation that non-uridylylated GlnZ interacts with AmtB (Huergo *et al.*2006a) and DraG forms a complex with GlnZ (Huergo *et al.*^2007^). The crystal structure of the *A. brasilense* DraG–GlnZ complex reveals an interaction surface on GlnZ opposite to that required for the GlnZ–AmtB interaction, invoking a structural model for the AmtB–GlnZ–DraG ternary complex (Rajendran *et al.*^2011^). A similar mechanism is likely to operate in other bacteria that possess the DraTG system. *Rhodospirillum rubrum* harbours three copies of genes encoding PII-like proteins, *glnB, glnJ and glnK*. Since *glnJ* and *amtB1* are co-expressed in this organism, GlnJ is likely to be the major player in terms of the interaction with AmtB1 and DraG. Although membrane localisation of DraG during ammonia switch-off is influenced by mutations in either *glnJ* or *glnB in vivo* (Wang *et al.*^2005^), the strongest association between AmtB1 and the PII proteins *in vitro* is observed with GlnJ (Wolfe, Zhang and Zhang 2007; Teixeira *et al.*^2008^).

The regulation of DraT and DraG activity in response to energy switch-off is less well understood. However, nitrogenase is post-translationally modified when *R. rubrum* is subjected to darkness or a decrease in oxygen concentration and such energy regulation of nitrogenase activity has been demonstrated in other diazotrophs (Hartmann and Burris 1987; Nordlund and Hogbom 2013; Zhang *et al.*^1997^). However, it is not yet known whether AmtB and the PII signal transduction proteins play a role in regulating DraG activity during energy depletion. Since the most highly conserved function of PII proteins is the binding of adenosine nucleotides and a role for these proteins in sensing the adenylate energy charge has been proposed (Jiang and Ninfa 2009; Forchhammer and Lüddecke 2015) it is feasible that DraG-PII interactions might also provide a mechanism for energy switch-off. However, there is evidence in photosynthetic bacteria that suggests that the mechanisms responsible for ammonia and darkness switch-off might be different. Notably, in contrast to ammonium-induced regulation of nitrogenase activity, GlnB and GlnJ remain uridylylated during darkness switch-off when cells are grown on dinitrogen as nitrogen source (Teixeira, Wang and Nordlund 2010). This implies that the PII proteins remain in the cytosol under these conditions and are not interacting with AmtB during energy depletion. It was also observed that the rate of nitrogenase inactivation varied according to the nitrogen source, being faster in dinitrogen grown cells compared with glutamate grown cells, perhaps indicating a difference in mechanism (Teixeira, Wang and Nordlund 2010). Moreover, AmtB is not required for darkness-dependent inactivation of nitrogenase in *Rhodobacter capsulatus* (Yakunin *et al.*^2001^) or anaerobiosis switch-off in *Azoarcus sp* BH72 (Martin and Reinhold-Hurek 2002), suggesting AmtB is only required for ammonium switch-off in these organisms.

Previous studies have identified DraG variants from *R. rubrum,* with altered regulatory properties. One of the most interesting of these is the N100K variant, which exhibits energy switch-off and modification of dinitrogenase reductase (NifH) in the dark but does not respond to ammonium switch-off (Kim, Zhang and Roberts 2004). The corresponding residue in *A. brasilense* DraG is D100, located in the interface with GlnZ in the DraG–GlnZ complex (Huergo *et al.*^2007^).

In this study, we have investigated the role of DraG membrane association in energy-dependent nitrogenase switch-off in *R. rubrum* and the potential role of AmtB proteins in this mechanism. In order to establish if the DraG location only is regulated under nitrogen-fixing conditions, we also monitored its location under non-diazotrophic conditions in both wild-type and AmtB mutants subjecting the cells to decreases in energy status as induced by darkness or oxygen limitation. We have further examined the role of the N100 DraG residue with respect to membrane sequestration and *in vitro* activity since this residue has been shown to be crucial for the *in vivo* activity of DraG during ammonium ‘switch-off,’ but not for energy related switch-off (Kim, Zhang and Roberts 2004).

## MATERIAL AND METHODS

### Strains and culture conditions

Wild-type *Rhodospirillum rubrum* S1 and mutants were grown in batch cultures in a minimal medium supplemented with N_2_/5% CO_2_ as the nitrogen source for nitrogen fixing (N-; Ormerod, Ormerod and Gest 1961) and 28 mM NH_4_^+^ (N+) for non-nitrogen-fixing conditions. Malate was used as carbon source in all conditions.

For the mutants, the medium contained antibiotics i.e. gentamycin (10 μg/ml) and/or kanamycin (50 μg/ml), respectively, as described earlier (Wang and Norén 2006). Strains are listed in Table 1.

**Table 1.** *R. rubrum* strains used.

Strains	Genotype and description	Reference
S1	Wild-type	http://genome.jgi.doe.gov/rhoru/rhoru.info.html
SNH-1	Insertional *amtB1*^−^, Gm^r^	Wang *et al* (2005)
SNH-3	Insertional double mutant *amtB1/2*^−^ Gm^r^, Km^r^	This work
UR212	*draTGB^−^ draT2:kan mutant, Km^r^*	Liang *et al.* (1991)
UR214	*draGB^−^ draG4:kan* mutant Km^r^	Liang *et al.* (1991)
SNH-4	Transconjugant of UR214 with *pUCGm, pGEX-6p-2 with N100S Quikchange substitution*	This work
SNH-5	Transconjugant of UR214 with *pUCGm w. pGEX-6p-2 with N100A Quikchange substitution*	This work
SNH-6	Transconjugant of UR214 with *pUCGm w. pGEX-6p-2 with N100K Quikchange substitution*	This work
SNH-7	Transconjugant of UR214 with *pUCGm w. pGEX-6p-2 with N100D Quikchange substitution*	This work

#### Anaerobic growth

Photosynthetic conditions were used during anaerobic growth and with either 28 mM NH_4_^+^ or nitrogen fixation using 95%N_2_/5%CO_2_ gas as nitrogen sources. Energy switch-off/on conditions were induced by subjecting the cultures to dark/light cycles.

#### Aerobic growth

During respiratory growth cells were grown in the dark with air and 28 mM NH_4_^+^ as nitrogen source. Energy ‘switch-off’ was induced by replacing the air atmosphere with 100% N_2_.

#### Microaerobic growth

To follow the effects of lowered oxygen concentration on nitrogenase activity and DraG location, we initially grew the cells under dark, aerobic conditions (see above) supplemented with 28 mM NH_4_Cl since *R. rubrum* cannot sustain initial growth under microaerobic nitrogen-fixing conditions in the dark. Cultures were grown to reach an OD_600_ between 1.0 and 1.5 for the wild-type, SNH-1 and UR212. For SNH-3, an OD_600_ between 0.7 and 1.2 was reached due to a slower growth rate. The cells were thereafter centrifuged and resuspended in the same minimal medium but replacing the NH_4_Cl with 2% O_2_/98% N_2_ gas to induce nitrogenase activity. The energy ‘switch-off’ was further performed by substituting the 2% O_2_/98%N_2_ with 100% N_2_.

### Activity assays

Nitrogenase activity was measured using the acetylene reduction method (Burris 1972).

#### In vivo nitrogenase switch-off assay

Two millilitres of samples were withdrawn from 500 ml cultures and incubated with 0.5 ml acetylene in the light at 30°C in 20 ml vials. Samples from the dark switch-off experiments were for each time point incubated for 2 min in the light before measuring the ethylene formed. Ammonium switch-off was induced by the addition of 10 mM NH_4_Cl to nitrogen-fixing cultures.

#### In vitro DraG activity

ADP-ribosylated dinitrogenase reductase (NifH) was incubated with purified DraG (10 μg) variants at 20°C and further analysed by SDS-PAGE and western blot (Berthold *et al.*^2009^).

### Construction of *R. rubrum* mutants

Construction of *amtB1* mutant (SNH-1) has been described in Wang *et al.* The double mutant *amtB1/amtB2* was constructed using a PCR-generated *amtB2* fragment cloned into pSUP202 vector giving *pSUP202::amtB2.* The *aphI* gene from pUC4K was excised with SalI and inserted into the partially SalI digested *pSUP202::amtB2.* Plasmids having the *aphI* gene in the same orientation as *amtB2* were isolated rendering pSNH002. To obtain a double mutant, pSNH002 was transferred into SNH-1 giving SNH-3 (*amtB1::aacC1,amtB2::aphI*) carrying Gm^r^ and Km^r^.

#### DraG N100 variants

DraG N100 substitutions were using PCR mediated site-directed mutagenesis (QuikChange, Stratagene, Agilemt, Kista Sweden) of *draG* giving: N100S, N100A, N100K and N100D. These substitutions were introduced into the fusion gene construct *GST-DraG* in pGEX-6p-2 in *Escherichia coli*, which was further subcloned into pUCGm. This plasmid was transferred into UR214 (*R. rubrum* DraG^−^) by conjugation with *E. coli* S-17, as described in Edgren and Nordlund (2004), resulting in DraG mutant *R. rubrum* strains SNH-4, SNH-5, SNH-6, SNH-7.

### Cell lysis and fractionation

Samples from all *in vivo* switch-off experiments were treated as described in Teixeira, Wang and Nordlund (2010), whereby cells were subjected to liquid nitrogen for 30 s before harvesting by centrifugation at 3000 × g for 10 min.

Cells were thereafter washed in 100 mM Tris-HCl buffer pH 7.8 and resuspended in 100 mM Tris-HCl pH 7.8 with EDTA-free protease inhibitors (Complete, Roche), DNase. Cell lysis was performed using a French Press at 18 000 psi or by lysozyme treatment (0.5 mg/ml) followed by sonication 4 × 30 s. Subcellular fractionation was carried out by centrifugation for 10 min at 11 000 × g to remove cell debri followed by 30 min 165000 × g spin. The chromatophore pellet was washed with 25 mM Tris-HCl pH 7.8. To release membrane-associated DraG, the pellet was resuspended in 0.5 M NaCl/25mM Tris-HCl pH7.8 and centrifuged at 165000 × g for 30 min. DraG localisation was analysed with SDS-PAGE followed by western blot.

## RESULTS

### Reversible DraG association to the chromatophore membrane depends on the energy status

According to our model, the activity of DraG is influenced by its reversible association with the chromatophore membrane during nitrogenase switch-off/on cycles. In order to investigate this further, lowered energy conditions were induced, either by imposing a light–dark cycle or by switching cells from microaerobic (2% O_2_) to anaerobic growth, while monitoring *in vivo* nitrogenase activity, dinitrogenase reductase (NifH) modification and DraG localisation during the alternating switch-off/on cycles.

**Figure 1.**
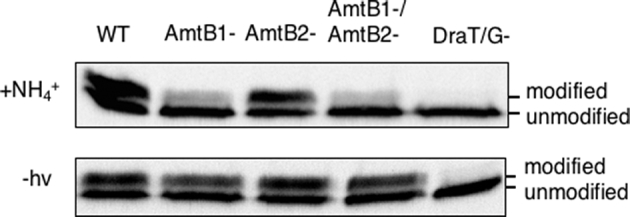
Effect of *R. rubrum amtB* mutations on post-translational modification of nitrogenase as monitored by *in vivo* NifH modification when cells were grown in phototrophic, nitrogen-fixing conditions and subjected to either ammonium shock (NH_4_^+^) or dark switch-off (–hv) conditions, respectively. (WT denotes the wild-type strain and DraT/G- *a draT::kan* insertion strain (UR 212), which is phenotypically DraTGB-^−^).

**Figure 2.**
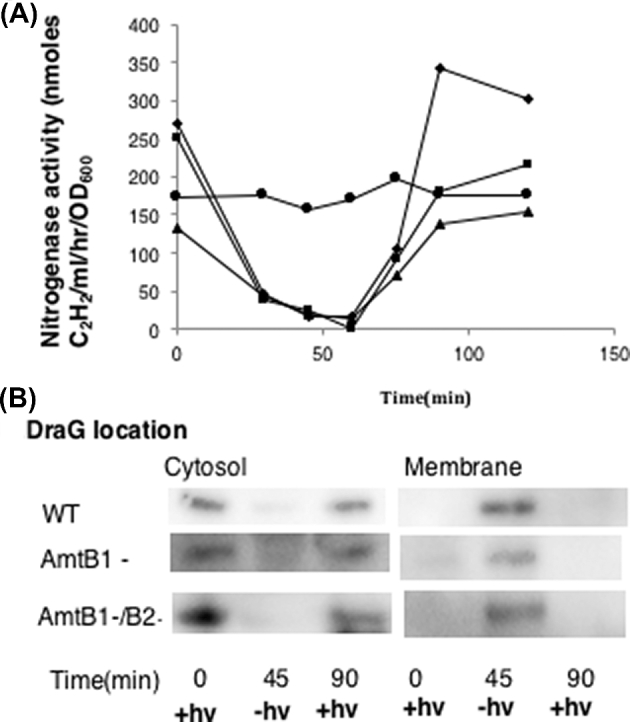
Correlation between the location of DraG with darkness switch-off of nitrogenase in phototrophic, nitrogen-fixing *R. rubrum*. (**A**) Nitrogenase activity measured by acetylene reduction in wild-type (diamonds), *amtB1* (squares) *amtB1, amtB2* double mutant (triangles) and the *draT::kan* insertion strain (UR 212), which is phenotypically DraTGB^−^ (circles). Darkness was imposed at time zero and light was restored after 60 min. (**B**) Location of DraG as determined by western blotting. The presence and absence of light is indicated by +hv and –hv, respectively.

Previous studies have demonstrated that *amtB1* is required for DraG to associate with the membrane under conditions of ammonium switch-off, but the potential role of *amtB2* was not investigated (Wang and Norén 2006). To examine the role of AmtB proteins in energy switch-off, we have utilised both single and double (*amtB1, amtB2*) mutant strains to investigate energy-related membrane sequestration profiles. Nitrogen-fixing cultures were grown photoheterotrophically under anaerobic conditions, subjected to either darkness or ammonium shock and subsequently assayed for covalent modification of dintirogenase reductase (NifH). A polar insertion mutation in *draT* (UR212), which fails to express DraT, DraG and DraB and perform reversible inactivation of nitrogenase, was used as a negative control (Liang *et al.*^1991^). As seen in Fig. 1 (top panel), following an ammonium shock, dinitrogenase reductase (NifH) was modified in the *amtB2* mutant. Further, neither AmtB1 nor AmtB2 were required for post-translational modification of nitrogenase when cultures were subjected to darkness (Fig. 1, bottom panel). In addition, we followed the kinetics of nitrogenase inactivation and reactivation during a light–dark cycle. Nitrogenase activity was rapidly inactivated upon the switch to darkness, irrespective of the presence of the single (*amtB1*) or double (*amtB1*, *amtB2*) mutations (Fig. 2A). The kinetics of nitrogenase inactivation was similar in the wild-type and *amtB* mutant strains, although the *amtB* mutants showed a slightly slower nitrogenase reactivation after light was reintroduced. As anticipated, nitrogenase activity did not alter significantly in the *draT* insertion mutant. In accordance with the switch-off of nitrogenase activity in the dark, a concomitant reversible change of the cellular location of DraG was observed, being located in the cytosol during light and membrane associated in the dark. The switch to the membrane during darkness occurred irrespective of the *amtB* mutations, suggesting that neither AmtB1 nor AmtB2 are required for the association of DraG to the membrane during darkness switch-off (Fig. 2B).

**Figure 3.**
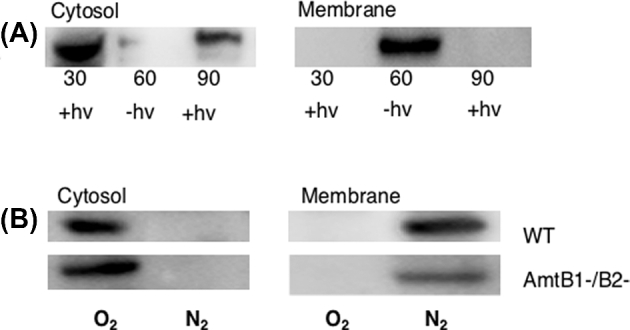
Effect of DraG location during ‘energy’ switch-off in ammonium grown *R. rubrum.* (**A**) Time course (in minutes) of DraG localisation in the *amtB1/B2*- double mutant during darkness switch-off as measured by western blotting. DraG exhibits reversible membrane association during the dark (–hv) light (+hv) cycle. (**B**) DraG locates to the membrane when dark grown cells are subjected to an energy shift after switching from aerobic (air) to anaerobic (100% N_2_) conditions. This occurs in the absence of both AmtB1 and AmtB2 (strain SNH-3). (WT denotes the wild-type strain).

Considering that DraG is a constitutively expressed protein and therefore not necessarily dedicated to the regulation of nitrogenase activity (Triplett, Wall and Ludden 1982), we were interested to determine if DraG locates to the membrane following a light-dark switch under non-nitrogen-fixing conditions. Cultures were grown with excess ammonium chloride (28 mM), which results in repression of nitrogen fixation (*nif*) gene expression and significant down-regulation of the *glnJ-amtB1* operon (Zhang, Pohlmann and Roberts 2005). Under these conditions, membrane sequestration of DraG occurred during the light–dark cycle, similar to that observed under diazotrophic conditions (Fig. 3a). This again suggests that membrane association of DraG following a switch to darkness is triggered independently of the nitrogen status and the presence of the AmtB proteins.

**Figure 4.**
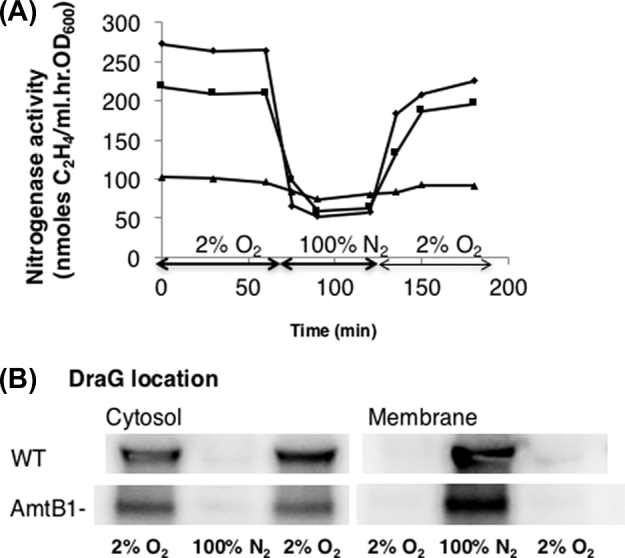
Correlation between nitrogenase activity and DraG location when strains are grown diazotrophically under microaerobic conditions in the dark (2% O_2_) and subjected to energy depletion (100% N_2_). (**A**) Time course of reversible energy switch-off of nitrogenase, as measured by acetylene reduction in wild-type (diamonds), *amtB1* (squares) and the *draT::kan* insertion strain (UR 212), which is phenotypically DraTGB^−^ (triangles). (**B**) Location of DraG during reversible switch-off in the wild-type or *amtB1* strain as determined by western blotting

To provide additional evidence that membrane association of DraG is regulated by the energy status, we examined nitrogenase switch-off under heterotrophic conditions when subjected to sudden oxygen depletion. Nitrogenase is an extremely oxygen sensitive protein *in vitro* but can tolerate low extracellular (microaerobic) concentrations of O_2_ that can be efficiently respired in the dark by *Rhodospirillum rubrum in vivo*. These conditions enabled us to investigate nitrogenase switch-off in response to an anaerobiosis shift. Cultures were grown in the dark under microaerobic conditions (2% O_2_), then shifted to anaerobic conditions (100% N_2_). This transition resulted in inactivation of nitrogenase until microaerobicity was restored, with concomitant reversible changes in the cellular localisation of DraG (Fig. 4B). As in the case of the light–dark switch, reversible inactivation of nitrogenase and membrane association of DraG were not influenced by the *amtB1* mutation, although slight differences were apparent in the rate of recovery of nitrogenase activity upon restoration of microaerobiosis (Fig. 4A).

To determine if this reversible membrane association also occurs in ammonium grown cells upon a respiratory switch, we grew cultures aerobically in the dark (20% O_2_), then subjecting them to anaerobic conditions by changing the gas phase to 100% nitrogen. This resulted in reversible membrane sequestration of DraG upon energy deprivation, independent of the presence of the *amtB1 and amtB2* mutations (Fig 3B). Thus membrane association of DraG induced by energy depletion is not dependent upon diazotrophic conditions or the presence of nitrogenase.

### The role of the N100 residue in *R. rubrum* DraG

**Figure 5.**
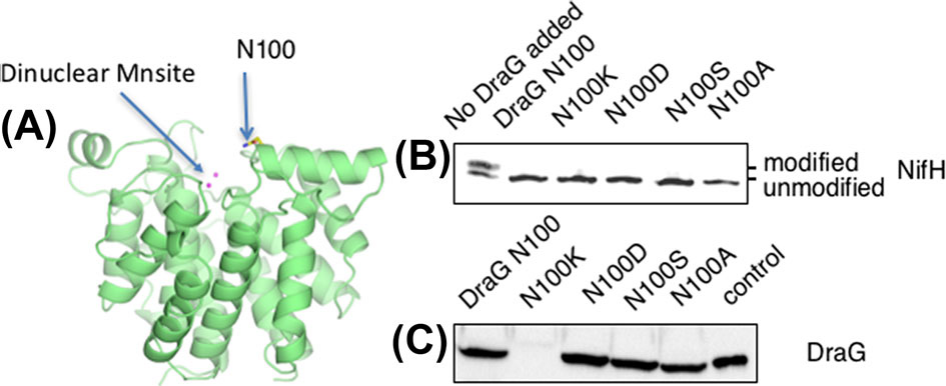
*In vitro* activity and cellular localisation of *R. rubrum* DraG in N100 substitution mutants. (**A**). Position of DraG N100 in the *R. rubrum* protein structure (pdb:2WOC). (**B)** DraG activity *in vitro* as monitored by NifH modification status using western blot analysis of NifH after incubation with purified DraG variants. (**C**) DraG location after ammonium shock using western blot analysis of the 0.5 M NaCl eluted membrane fractions from fractionated extracts of *R. rubrum* strains expressing the indicated DraG variants. As a control 0.5 M NaCl eluted DraG from wt *R. rubrum* membranes was used.

As seen in Fig. 5A, the N100 residue is located in the vicinity of the dinuclear manganese active site and close to the surface of the *R. rubrum* DraG structure (Berthold *et al.*^2009^). The N100K substitution in *R. rubrum* DraG is predicted to disrupt the interaction with GlnJ R103, hence explaining the defect in ammonium switch-off (Rajendran *et al.*^2011^). In order to obtain a more detailed understanding of the role of N100 in DraG function, we generated three additional substitutions at position 100 with different charge properties to elucidate the effect on *in vivo* membrane interaction and *in vitro* dinitrogenase reductase (NifH) demodification. The variant DraG proteins were overexpressed and purified from *Escherichia coli* as GST-tagged fusions, and their activities were assayed *in vitro* by mixing ADP-ribosylated dinitrogenase reductase (NifH) with DraG. Samples were withdrawn for western blot analysis to determine the extent of dinitrogenase reductase (NifH) demodification. All of the N100 substitutions catalysed the removal of the ADP ribosyl group from dinitrogenase reductase (NifH), indicating that all of them retained the catalytic function of DraG (Fig. 5B). To determine the influence of the N100 residue on membrane association, wide-host range plasmids expressing the DraG variants were introduced into *R. rubrum* strain UR214 (*draTGB-*). Reversible membrane interaction by the DraG variants was monitored in photo-heterotrophically grown diazotrophic cultures subjected to ammonium switch-off, and of the 4 variants constructed, the N100K variant exclusively lost its ability to associate with the membrane, while the other substitutions at position 100 still interacted (Fig. 5C). This confirms that introducing a positive charge at this position interferes with the membrane association during ammonium switch-off with a loss of regulation.

## DISCUSSION

A number of studies have shown that inactivation of DraG activity is due to sequestration to the inner membrane in *Rhodospirillum rubrum* and *Azospirillum brasilense*, the two best-studied diazotrophs with respect to the metabolic regulation. Taken together, our results suggest that the mechanism by which DraG associates to the membrane is clearly different when comparing the response to ammonium shock with energy depletion and suggests that the binding targets for DraG are different in each case.

As indicated by studies on *A. brasilense,* interaction between DraG and PII proteins could provide a mechanism for nitrogen control of DraG activity through the formation of DraG: PII complexes (Huergo *et al.*^2007^; Rajendran *et al.*^2011^). In accordance with this, under ammonium switch-off conditions, membrane association of DraG in *R. rubrum* requires AmtB1 and either GlnJ or GlnB (Wang *et al.*^2005^).

In this study, we have verified that AmtB1 is required for post-translational modification of nitrogenase under ammonium-induced switch-off conditions (through inactivation of DraG), but the second ammonium transporter in *R. rubrum*, AmtB2, does not appear to be involved in the signalling mechanism.

The DraG N100K substitution, which is predicted to perturb the interaction with GlnJ, results in an altered switch-off response to ammonium but not to darkness (Kim, Zhang and Roberts 2004). To discriminate if this results from an effect on DraG enzyme activity or its localisation, we assayed the *in vitro* activity of DraG N100K and three other substitutions of N100 and examined their ability to associate with the membrane following ammonium shock. In all cases DraG was active *in vitro* but, in contrast to the other variants, the N100K substitution did not localise to the membrane *in vivo*, supporting the prediction that this substitution influences the interaction with the membrane, whereas the N100A, N100D and N100S substitutions are unlikely to disfavour association via charge repulsion. The absence of membrane association in the N100K variant therefore supports a model in which DraG is normally inactivated when interacting with GlnJ–AmtB1 following ammonium shock. However, this is unlikely to be the case under energy-depleted conditions, firstly, because AmtB1 is not required for membrane sequestration during energy switch-off and secondly, the N100K substitution does not influence switch-off during the dark (Kim, Zhang and Roberts 2004), implying that DraG is likely to interact with a different binding partner during energy-depleted conditions.

A difference in the response between the two effectors (ammonium and energy status) switch-off was initially observed in *A. brasilense*, whereby an *ntrBC* mutant strain exhibited altered switch-off in response to ammonium but switch-off in response to anaerobiosis was not affected (Zhang *et al.*^1994^). As NtrC controls the expression of AmtB in *A. brasilense*, these results imply a divergent route for the signal transduction pathway of energy dependent DraTG regulation. Similarly, an *A. brasilense* GlnZ mutant behaved differently in response to ammonium and energy switch-off stimuli, reflecting the involvement of PII signal transduction proteins in ammonium, rather than energy status switch-off (Klassen 2001). The studies we report here show that *R. rubrum* DraG is bound to the membrane when the energy level is reduced under either photosynthetic or respiratory conditions. Surprisingly, reversible membrane sequestration of DraG occurs regardless of whether the cells are grown diazotrophically or non-diazotrophically. Hence, changes in energy status, possibly reflecting alterations in membrane polarisation, influence the localisation of DraG, independently of nitrogen fixation. This again strongly suggests that AmtB1 and GlnJ are not required for membrane localisation of DraG in response to the energy status, as both proteins are poorly expressed under ammonium-rich conditions. This is further supported by the finding that DraG is still regulated by membrane sequestration upon energy-related switch-off in *R. rubrum amtB* mutants, regardless of nitrogen-fixing conditions.

Taken together, we suggest a model in which DraG binds to a specific membrane protein that is affected by changes in the energy status in the cell. The signal from energy metabolism is not likely to be a consequence of changes in the ATP level, as this does not change dramatically when *R. rubrum* is subjected to darkness (Lindblad and Nordlund 1997). On the other hand several studies have shown that the NAD^+^/NADH ratio increases significantly when *R. rubrum* cultures are subjected to darkness (Klamt *et al.*^2008^; Jackson and Crofts 1968; Carius, Rumschinski and Faulwasser 2014). It is therefore more likely that interactions between DraG and a membrane protein is affected by sensing changes in the redox status or possibly the membrane potential.
